# Personalizing Medicine and Technologies to Address the Experiences and Needs of People with Multiple Sclerosis

**DOI:** 10.3390/jpm11080791

**Published:** 2021-08-12

**Authors:** Adam Henschke, Jane Desborough, Anne Parkinson, Crystal Brunoro, Vanessa Fanning, Christian Lueck, Nicola Brew-Sam, Anne Brüstle, Janet Drew, Katrina Chisholm, Mark Elisha, Hanna Suominen, Antonio Tricoli, Christine Phillips, Matthew Cook

**Affiliations:** 1 Department of Philosophy, Faculty of Behavioural, Management, and Social Sciences, University of Twente, 7522 Enschede, The Netherlands; 2 Crawford School of Public Policy, College of Asia and Pacific, Australian National University, Canberra 2601, Australia; 3 Department of Health Services Research and Policy, Research School of Population Health, College of Health and Medicine, Australian National University, Canberra 2601, Australia; jane.desborough@anu.edu.au (J.D.); anne.parkinson@anu.edu.au (A.P.); crystal.brunoro@anu.edu.au (C.B.); vfanning29@gmail.com (V.F.); Nicola.Brew-Sam@anu.edu.au (N.B.-S.); janet.drew@anu.edu.au (J.D.); katrina.chisholm@bigpond.com (K.C.); markelisha2@gmail.com (M.E.); 4 Australian National University Medical School, College of Health and Medicine, Australian National University, Canberra 2601, Australia; Christian.lueck@act.gov.au (C.L.); christine.phillips@anu.edu.au (C.P.); 5 Department of Neurology, Canberra Health Services, Canberra 2605, Australia; 6 John Curtin School of Medical Research, College of Health and Medicine, Australian National University, Canberra 2601, Australia; Anne.Bruestle@anu.edu.au (A.B.); matthew.cook@anu.edu.au (M.C.); 7 School of Computing, College of Engineering and Computer Science, Australian National University, Canberra 2601, Australia; hanna.suominen@anu.edu.au; 8 Machine Learning Group, Data61, Commonwealth Scientific and Industrial Research Organisation, Marsfield 2122, Australia; 9 Department of Computing, Faculty of Technology, University of Turku, 20500 Turku, Finland; 10 Research School of Chemistry, College of Science, Australian National University, Canberra 2601, Australia; antonio.tricoli@anu.edu.au

**Keywords:** personalized medicine, multiple sclerosis, lived experience, uncertainty, fatigue management

## Abstract

There is enormous variation in the manifestations of disease experienced by people with multiple sclerosis (PwMS). While this variation makes personalized medicine an attractive goal, there are many challenges to be overcome before this opportunity can be realized. Personalized medicine often focuses on targeted therapies and detailed monitoring, but we also need to recognize that there will be variation in acceptance of these approaches by different PwMS. In other words, deep personalization of medicine will encompass targeted therapy, precision monitoring, tailored to variation in personal attitudes to these transformations in health care. In order to meet the promise of personalized medicine for MS, understanding the experiences of PwMS is necessary both to aid in the uptake of personalized medicine, and to ensure that personalized approaches to monitoring disease and treatment provide a net benefit to PwMS rather than placing additional burdens and stressors on them. Here, we describe recent research that identified five experiential themes for PwMS, and then interpret these themes according to the foundations of personalized medicine to provide a road map for implementation of personalized medicine solutions for PwMS.

## 1. Main Manuscript

It is 20 years since the near-complete draft sequence of the human genome was released. Now that it is feasible to re-sequence human genomes quickly and cheaply, personalized medicine stands out as the most immediate and tangible consequence of the human genome project on clinical practice. Advances afforded by genomics have been complemented by other technological developments that provide opportunities for near-continuous monitoring of fluctuations in phenotype and environmental variables. When combined with precision therapies implemented according to patient stratification by mechanism of disease, these advances provide the opportunities for the transformation to personalized medicine.

Precision medicines describe the use of therapeutics that target a specific molecule, cell or pathway, exemplified by monoclonal antibodies or recombinant receptors. Personalized medicine is when such drugs are selected for use in patients most likely to benefit, based on knowledge of the pathophysiological pathway responsible for their disease and the expected individual response to that drug. This concept is very clear in MS [1,2,3], for which there are many approved precision therapies, but the main obstacle to better clinical outcomes is timely selection of the most appropriate precision therapy for any particular patient.

**Figure 1:**
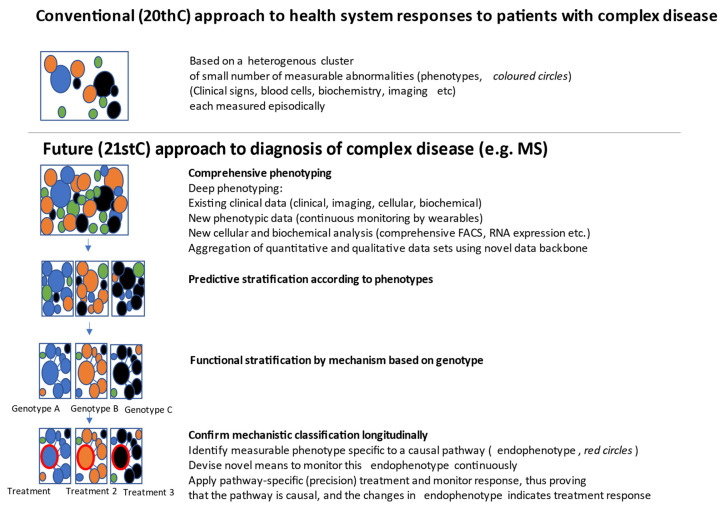
How personalized medicine will change the understanding and treatment of complex conditions such as MS.

While considerations of individual genomic variation that might inform mechanism of disease and risks of side effects from treatments (pharmacogenomics) are the cornerstones of personalized medicine, we are also at a juncture where the increased capacity for personalization of medicine is set to transform medicine and health care more broadly, as outlined in Figure 1.

Simultaneously, advances in data science and analytics, enabled by electronic health records, networked information systems, artificial intelligence, and machine learning, among other computational methods, have been prevalent, if not disruptive technologies, and have catalyzed rapid transformations [4,5,6]. Consequently, personal devices are used increasingly to monitor physiological variables, and with societal changes in attitudes to consent and authority within the health sphere, deep personalization of health care will see individuals taking greater control over both collection and access to their health and medical data. In other words, increasingly, we have access to methods and devices that can increase patient participation in maintenance of health, pursuit of timely treatment, involvement in research, and contribution to priorities in health care, but this has implications for patients’ experience of health care.

A recent metasynthesis of people’s experiences of living with MS [3] identified five key themes that describe these experiences: (1) the quest for knowledge, expertise, and understanding, (2) uncertain trajectories, (3) loss of valued roles and activities, and the threat of a changing identity, (4) managing fatigue and its impacts on life and relationships, and (5) adapting to life with MS. This paper seeks to show the value of identifying and understanding these themes, in order to ensure that personalized medicine responds to the experiences and needs of PwMS. These themes are outlined in Table 1 and Table 2, described and their relevance to personalized medicine are then explained.

### 1.1. The Quest for Knowledge, Expertise and Understanding

People diagnosed with MS commonly embark on self-directed research in an effort to understand their condition. This can be a confronting exploration as they learn that the fundamental cause of MS remains unknown, manifestations of MS vary from person to person, and this variability translates into uncertainty about prognosis and responses to treatment. Patients and clinicians face a bewildering choice of drugs. While demonstration of clinical efficacy in randomised trials is usually required for approval and funding of drugs, a statistically significant result in a randomised clinical trial is of course difficult to translate into information for the individual in the consulting room. Furthermore, most drugs carry risks of adverse events, which are usually infrequent but unpredictable. Finally, there is no consensus on the efficacy or effectiveness of lifestyle modifications such as dietary approaches, exercise, rehabilitation, and stress management. Indeed, the information available is often conflicting.

At present, and for the foreseeable future, personalized medicine will be characterized by uncertainty. This arises, at least in part, due to the sheer scale of datasets that form the building blocks for personalized medicine. As more technologies are able to gather information about the condition, treatments, and efficacy of treatments from a range of sources, and as data science and analytics improve the integration of this information into practically usable knowledge, better health care should be the result. In the short term, however, the magnitude of data with which PwMS are confronted renders them incomprehensible. This is a potential obstacle to engagement of PwMS in the goals and potential benefits of personalized medicine. For example, if PwMS can see no clear connection between the information being gathered and improvement in their treatment, outcomes or general understanding, this will ultimately undermine ongoing use and support of the relevant technologies. Furthermore, there is a risk that responsibilities for data collection by PwMS may become an additional burden on top of the illness itself.

### 1.2. Uncertain Trajectories

People with MS are deeply affected by the uncertainty and perceived loss of control associated with the disease. The extent to which, for example, loss of mobility, pain, fatigue, cognitive changes, or bladder and bowel symptoms may impact on their activities and social participation day-by-day is unpredictable. In the absence of an accurate prognosis, major life decisions such as career choices and family planning become a fraught exercise. Most PwMS experience profound anxiety about the long-term future progression of their disease as well as the potential loss of independence.

In addition to the burden of gathering data about their own illness, the information itself can be overwhelming (“data-whelming”) and reinforce increased uncertainty about prognosis and responses to treatment. Large volumes of information, particularly information that remains uninterpretable, can produce greater uncertainty rather than greater knowledge. MS is a highly variable condition and increasing the information available to PwMS will not necessarily reduce uncertainty. Instead, information needs to be both useful for PwMS, and managed in a way that is flexible and adaptable so that the scale and complexity of datasets can be tailored to different PwMS according to their expectations, needs, and treatments.

### 1.3. Loss of Valued Roles and Activities and the Threat of a Changing Identity

An MS diagnosis can create a sense of grief and loss [3]. This may be related to many factors, including the threat to independence, uncertainty about capacity to continue to work and/or to care for family, maintain physical strength, and continue with cherished non-work-related interests. Often, PwMS feel that their identity is impacted and that others may perceive or relate to them differently. Identity and information, particularly personal information that relates to health care and medical treatments, stand in a relationship where each changes the other [7]. Receiving a diagnosis of MS changes how a person understands themself and the way they gather and process information. Of course, the magnitude of these effects varies from person to person. Similarly, PwMS have different responses to uncertainty, and this translates into different levels of engagement with what are sometimes complex and sophisticated concepts when engaging with personalized medicine.

### 1.4. Managing Fatigue and Its Impacts on Life and Relationships

When we consider the deep personalization of medicine, we should not forget an enduring principle of medicine; that the patient is the expert on their own symptoms. In this sense, the clinician has to come to terms with uncertainty and variability in how patients’ experience their physiological cues. One of the most commonly reported and troubling symptoms for PwMS is fatigue [3]. Fatigue is hard to physically measure, is a relatively contested term with multiple potential meanings [8], and may be physical, emotional or cognitive, either alone or in combination. The medical causes and treatment of MS-related fatigue remain poorly understood and currently no medications are regarded as effective for MS fatigue. While non-pharmacological interventions show some promise, they are under-investigated and robust evidence for their efficacy is lacking [9]. The burden of fatigue experienced by PwMS is often compounded by the lack of community understanding about MS-related fatigue, manifesting as negative perceptions of the validity of their symptoms—both at home and at work.

Objective data from personalized technologies might help to offset these difficulties. For example, if they can signal to the PwMS when they might expect to have a day affected by fatigue, and enable them to put in place management strategies, and signal to family, friends, carers, or clinicians that they are likely to experience a day affected by fatigue. Of course, such a process would also need to adhere to privacy conditions—that personal disclosures would need to be chosen by the PwMS.

There is also the potential that fatigue might exacerbate data-whelming, particularly if data gathering requires concerted effort. If data gathering is a manual activity, it is likely to be less complete on days when PwMS are affected by fatigue. Furthermore, data gathering might contribute to fatigue and hence, the perceived ease and effortlessness of use by PwMS must be considered as a central design principle in related technology development and evaluation (see our further elaboration below). Given the significant variability of living with MS, effective personalized medicine needs not only to understand and flag fatigue, but also to be responsive to individuals’ varying fatigue states.

### 1.5. Adapting to Life with MS

PwMS develop and draw on various strategies to adapt to living with a chronic disease. These may include the use of assistive technologies, deriving support from peer groups, other community members, spiritual resources or networks, and learning to redefine their identity through re-orienting their professional and social way of life around their changed reality.

The extreme heterogeneity of manifestations of MS, variation in symptom severity, plus differing but unpredictable rates of disease progression, offers potential for the development of person-specific approaches to treatment, and monitoring of the outcomes of these treatments. Only a personalized approach will enable a combination of pharmacological and non-pharmacological interventions that result in amelioration of symptoms, halt the progression of disease, or even reverse central nervous system damage. Equally, effective and robust outcome measures rely on a personalized approach to monitoring and measurement. Here, we emphasize that personalized medicine needs to employ personalizable technologies. In other words, the overall objective of personalized medicine is to assist people in adapting to life with MS, including their interactions with family, friends, carers, and clinicians. However, in order to achieve this objective, technologies and their interfaces need to take into account the aforementioned five themes: (1) the quest for knowledge, expertise, and understanding, (2) uncertain trajectories, (3) loss of valued roles and activities, and the threat of a changing identity, (4) managing fatigue and its impacts on life and relationships, and (5) adapting to life with MS, and be customizable and dynamic.

These experiential themes identified in the metasynthesis [3] provide important insights into the concerns and needs of PwMS. Through mapping these themes to current concepts of personalized medicine, we can gain a better understanding of what is necessary to develop personalized medicine solutions for PwMS. By understanding and acknowledging what PwMS experience, we can also identify the particular benefits that personalized medicine may offer PwMS.

### 1.6. Personalized Medicine Addressing Uncertainty

Personalized medicine may have a role in alleviating uncertainty about disease trajectories in the future. At present, PwMS and their clinicians are confronted with many immunotherapeutics. PwMS may have a preference for the route of administration (for example, pill vs. injection) but effectiveness can only be determined retrospectively. Thus, deciding on which treatment to use for a given individual is problematic [10].

Treatment failure is often perceived by PwMS when they develop new symptoms, and then confirmed by changes detected on Magnetic Resonance Imaging (MRI). An important objective of personalized medicine and close monitoring of PwMS is to lower this “detection threshold” and identify failure of treatment earlier based not only on symptoms but easily measured and reliable biomarkers of disease activity. For example, detection of neurofilament light chain (NfL), a breakdown product of damaged neurons, has become widely accepted as an indicator of new MS activity, independent of the area of the nervous system affected [11,12]. Such a biomarker could lower the detection threshold for disease progression, potentially in the absence of change in symptoms.

Progress is likely to come in small steps, with enhanced detection methods and additional biomarkers to lower the threshold, as well as better prediction of risks of adverse events through pharmacogenomics that can identify individual differences in drug absorption and metabolism. We expect this will enable refinement of drug choice and dosage.

### 1.7. Personalized Medicine Improving Diagnosis and Monitoring

Personalized approaches to MS diagnosis, including the development of multivariate predictive diagnostic and prognostic models, which incorporate biomarkers and environmental exposures, could assist in improved and earlier diagnosis [13]. These approaches also offer the possibility for developing and improving methods to develop a “risk score” for MS [14]. Furthermore, identification of accurate MS biomarkers of disease progression and treatment response that can be monitored by non-invasive devices could reduce uncertainty faced by PwMS throughout their illness. Data from personal devices (‘wearables”, “wearable sensors” or “wearable sensor technologies”), particularly smartphones [15], might be linked with information from point-of-care or portable devices to monitor MS biomarkers affordably, frequently, and with minimal discomfort. Frequent monitoring of biomarkers, such as NfL, is a natural complement to precision medicine, as it will provide early indication of treatment efficacy and potentially adverse effects, and therefore reduce uncertainty. These “big data” would enable combining and comparing a person’s data holistically across their sources and in time, thereby powering predictive systems and recovery from scenarios where, for example, a problem was not captured by pathology tests or other measurements taken as part of a given health care appointment but rather earlier recordings from home. One approach is to use this rich personal information generate “digital twins” for PwMS, an informational analogue to the real-world person [16]. We note here that such virtual identities pose significant ethical concerns which must be dealt with in the design, application, and use of these digital twins [7].

### 1.8. Personalized Medicine Reducing the Risk of Treatment Mistakes

While personalized approaches to monitoring of disease progression should assist clinicians and PwMS to decide about treatment, it is unlikely that uncertainty will be eliminated completely. Indeed, personalized medicine depends on the collection of very large data sets, and as most of these data points will go uninterpreted, overall uncertainty will likely rise [17]. Personalized medicine encompasses prognostication and treatment decisions based on complex datasets including genomics, which risks rendering decisions even more difficult for both clinicians and patients. We need to be able to identify and extract “crucial” information, even if it represents only a small proportion of these large datasets. In this way, we can begin to take the steps necessary to reduce the current level uncertainty faced by PwMS. We must also empower PwMS to participate in clinical judgement and decision-making related to their own treatment, for example, by personalized data science and analytics technologies to serve their needs [18,19].

### 1.9. Implications of Personalized Medicine for the Health Care System-Feedback, Co-Design and Implementation

Personalization needs to be seen as a dynamic relationship between PwMS, their health care providers, and the larger clinical system supporting them. This means taking advantage of available technology to empower patients to provide feedback to their clinicians about their progress—whether it is the incidence and intensity of fatigue, or effectiveness and adverse effects of treatments. Feedback is essential to ensure personalization of diagnoses and treatments for MS, which varies so significantly from person to person and through time.

People who have MS are potential adopters of personalized medicine—as Griffin and Kehoe note, PwMS are both high users of smartphones, and generally see smartphones as beneficial and useful [15]. It is likely that personalized medicine will challenge existing medical practices and have implications for clinician knowledge and the experiences of PwMS as they navigate a complex health system. Equally, substantial challenges for clinicians are inevitable as they are confronted with growing and complex datasets about biological variables, environmental exposures and lifestyle decisions of their patients [20]. They will need tools to transform and translate this information to inform decisions about treatment. At the same time, PwMS will be in a position to provide their clinicians with rich accounts of their lived experience with MS. Readying health care organizations for these transformations will extend beyond clinicians to policy-makers and service leaders, and will demand appropriate capacity building to deal with this information [21]. Thus, in order to ensure that personalized medicine is a good value proposition for all, in addition to patients, clinicians and health services need to be involved in the design, conduct, and evaluation of personalized medicine research and implementation. Such strategies to enable more seamless care in view of increasingly complex health practice dynamics and information will be needed for a truly patient-centered era of personalized medicine.

## 2. Conclusions

The combination of pharmacological and non-pharmacological interventions that may bring about an amelioration of the symptoms in each patient or result in a halt in the progression of the disease, or even a reversal of central nervous system damage, is almost certainly only going to be possible using a personalized approach. Equally, effective and robust outcome measures must rely on a personalized approach to monitoring and measurement. The development of new tools such as wearables, data analytics, smartphones and NfL to provide an earlier diagnosis of MS, and the increased knowledge gained from bioinformatics, offer significant hope for improved diagnosis and treatment for MS [15,22]. However, to ensure that the promises of personalized medicine for MS are realized, we must engage with PwMS’s lived experiences, to develop and adapt personalized approaches such that they are actually personalizable. For a complex condition such as MS, which presents in such individual ways, personalized medicine and related technologies must be truly personalized.

## Figures and Tables

**Table 1:** A summary of issues of concern for PwMS [3] and related elements for consideration in the development of personalized medicine and technologies.

Themes [3]	Concerns Raised by PwMS [3]	Key Elements for Consideration
1. Quest for knowledge, expertise and understanding	The ability of PwMS to make decisions is hampered by a lack of knowledge about the efficacy and suitability of disease modifying treatments.	Electronic health technologies that monitor and capture individual disease behaviour may compensate for limitations of access to relevant services. PwMS need to see a clear connection between information being gathered and improved treatments and outcomes resulting from personalized medicine. More information is not necessarily better; it can be overwhelming and act as an obstacle to engagement. Practice and technological design need to be able to respond when people’s desire for more or less information changes to protect against information overload.
2. Uncertain trajectories	Unpredictability of symptoms on a day-to-day basis inhibits planning. Uncertainty of disease trajectory impacts life decisions. Many PwMS experience profound anxiety about long-term future progression and potential reduced independence.	Better prediction of risks of adverse events through pharmacogenomics that can identify differences in drug absorption and metabolism, guiding drug choice and dosage. Identification of disease biomarkers for improved monitoring of disease progression and treatment response, combined with development of non-invasive devices to detect these biomarkers, could address the uncertainty PwMS face throughout the illness trajectory. Feedback from patients using available technology is an essential aspect of ensuring the personalization of diagnoses and treatments.
3. Loss of valued roles and activities and the threat of a changing identity	Receiving an MS diagnosis changes how a person understands themselves and their identity PwMS will have different responses to uncertainty. PwMS will understand the same information differently PwMS will have different needs for information.	Personalized medicine must be responsive to people’s different and changing through the course of their diagnosis and ongoing treatment.
4. Managing fatigue and its impacts on life and relationships	PwMS report fatigue impacts their lives greatly. Fatigue is poorly understood with no current effective medications. Non-pharmacological interventions are under investigated and lack robust evidence.	Given the significant variability of living with MS, any effective personalized medicine needs not only to understand and flag fatigue, but also to respond to individuals’ varying fatigue states.
5. Adapting to life with MS	PwMS develop and draw on various strategies to adapt to living with MS. Many PwMS learn to redefine their identity through re-orienting their professional and social way of life around their changed reality.	Only a personalized approach will enable a combination of pharmacological and non-pharmacological interventions that may result in an amelioration of symptoms, a halt in the progression of the disease, or even a reversal of damage to the central nervous system. Sufficiently frequent monitoring of MS biomarkers via non-invasive devices has great potential to both address uncertainty faced by people with MS, as well as to direct and monitor precision medicine treatment efficacy.

PwMS: People with multiple sclerosis.

**Table 2:** A summary of the implications of personalized medicine and technologies for the health care system.

Key Areas for Consideration	Potential Solution
Personalization needs to be a dynamic relationship between the patient and their direct health care providers as well as the larger clinical system supporting them.	Use available technology to empower patients to provide feedback to their clinicians about their progress, e.g., incidence/intensity of fatigue, monitoring of effectiveness, and side effects of treatments. Feedback is an essential aspect of ensuring the personalization of diagnoses and treatments for a heterogeneous condition such as MS.
People who have MS and health care providers are the proposed adopters of personalized medicine. It is likely that personalized medicine will challenge existing medical practices and have implications for clinician knowledge and the patient experience, especially in navigating an already complex health system.	Clinicians will need to make decisions spanning increasingly complex biological, environmental and lifestyle information, as well as translate this information into meaningful information for patients considering their treatment options.
Readying health care organizations for change extends beyond clinicians, to include policy and service leadership and capacity.	To ensure that personalized medicine is a good value proposition for all, in addition to patients, clinicians and health services need to be involved in the design, conduct, and evaluation of personalized medicine research and implementation.
Personalization of MS treatments future efforts in the development of technologies that can quantify the impact on disease manifestation and progress.	To optimize uptake and sustainability in clinical practice, PwMS and clinicians need to be involved in the design, implementation, and evaluation of personalized technologies.

## Patient and Public Involvement

In the true spirit of patient and public involvement, our research team includes six people living with MS, four of whom have contributed to this paper.
